# Supplementary material for: Sylvatic Canine Morbillivirus in Captive Panthera Highlights Viral Promiscuity and the Need for Better Prevention Strategies
Source: Pathogens. 2021 Apr 30;10(5):544. doi: 10.3390/pathogens10050544 (PMC8147164; doi:10.3390/pathogens10050544)
Supplement: Supplementary file 1 [file pathogens-10-00544-s001.zip › pathogens-1179065-supplementary.pdf]

Table 1. Detailed signalment of felids with confirmed vaccination previous to the outbreak in 2015–2016. Per confirming records, all felids have been vaccinated with Nobivac Puppy-DPv (modified live vaccine that covers canine distemper and parvoviruses). n.a. = not applicable.

Species	Sex	Arrival to the Center (year)	Vaccination Date	Vaccinated Date(s) during the Outbreak
lion	f	2012	24/03/2013	31/10/2015 and 3/12/2015
tiger	m	1998	12/06/2000	3/12/2015
tiger	m	2011	28/09/2012	31/10/2015 and 3/12/2015
tiger (case#4)	f	2010	25/03/2013	n.a.
tiger	m	2010	23/03/2013	31/10/2015 and 3/12/2015
lion	f	2012	24/03/2013	1/11/2015
lion	f	2005	23/04/2009	1/11/2015
leopard	m	2006	26/03/2009	1/11/2015
tiger	f	2004	3/12/2014	1/11/2015
leopard	m	2006	3/12/2014	1/11/2015
tiger	f	2013	9/04/2013	1/11/2015 and 20/11/2015
tiger	f	2000	05/09/2009	1/11/2015
tiger	m	2006	31/3/2006	1/11/2015
tiger	m	2006	31/3/2006	1/11/2015
tiger	m	2006	31/3/2006	1/11/2015 and 5/12/2015
tiger	f	2009	19/07/2009	1/11/2015 and 5/12/2015
tiger	f	2009	19/07/2009	1/11/2015 and 5/12/2015
tiger	m	2010	27/09/2010	1/11/2015
tiger	f	2010	27/09/2010	5/12/2015
leopard	m	2010	27/09/2010	1/11/2015 and 5/12/2015
tiger	m	2009	13/03/2013	1/11/2015 and 5/12/2015
lion	m	2010	27/09/2010	1/11/2015
tiger	m	2011	28/09/2012	1/11/2015
tiger	f	2009	19/07/2009	1/11/2015
tiger	m	2006	22/08/2006	1/11/2015
tiger	m	2006	22/08/2006	1/11/2015
tiger	m	2006	22/08/2006	1/11/2015
lion	f	2012	24/03/2013	1/11/2015
lion	m	2012	18/10/2012	1/11/2015
bobcat	m	2010	3/12/2014	n.a.
asian leopard	m	2007	16/03/2009	n.a.
lion	m	2000	23/05/2013	1/11/2015
lion	f	2000	28/08/2000	1/11/2015

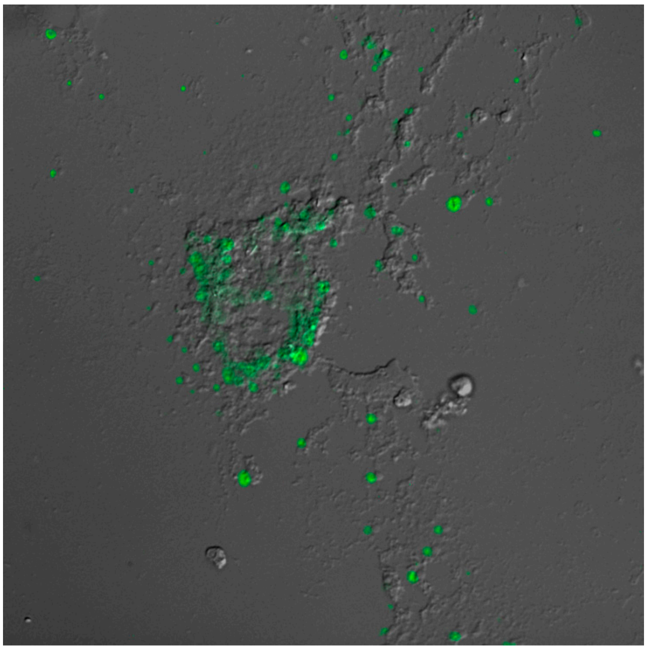

Figure S1. Direct immunofluorescence assay from lung from tiger #3. Bright green signal correspond to canine distemper epitopes.

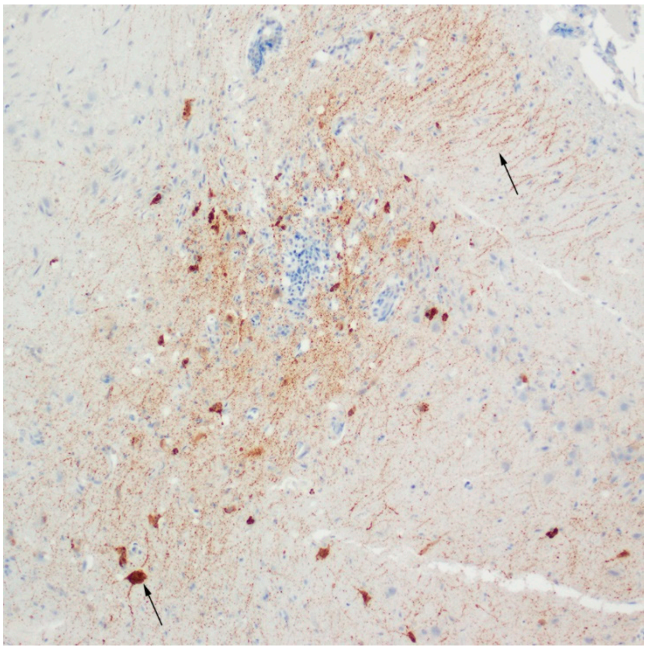

Figure S2. Immunohistochemistry for CDV from brain from a raccoon from IN harvested in May of 2016 approximately 2.5km from the rescue centre: There is strong immunoreactivity to CDV-epitopes that accumulate within axons and perikarya; in some areas the nucleus is obscured (arrows). Unfortunately no CDV-genetic material was available for comparison of the H-gene sequences.
